# Inhibitory effects of tanshinones towards the catalytic activity of UDP-glucuronosyltransferases (UGTs)

**DOI:** 10.3109/13880209.2015.1045621

**Published:** 2017-05-03

**Authors:** Xu-Xin Zhang, Yun-Feng Cao, Li-Xuan Wang, Xiao-Lin Yuan, Zhong-Ze Fang

**Affiliations:** a Affiliated Zhongshan Hospital of Dalian University, Dalian, China;; b Joint Center for Translational Medicine, Dalian Institute of Chemical Physics, Chinese Academy of Sciences and The Affiliated Zhongshan Hospital of Dalian University, Zhongshan District, Dalian, China;; c Key Laboratory of Contraceptives and Devices Research (NPFPC), Shanghai Engineer and Technology Research Center of Reproductive Health Drug and Devices, Shanghai Institute of Planned Parenthood Research, Shanghai, China;; d Joint Center for Translational Medicine, Dalian Institute of Chemical Physics, Chinese Academy of Sciences and First Affiliated Hospital of Liaoning Medical University, Dalian, China;; e Department of Toxicology, School of Public Health, Tianjin Medical University, Heping District, Tianjin, China

**Keywords:** Enzyme inhibition, herb–drug interaction, *in vitro* screening, *in vitro–in vivo* extrapolation (IV-IVE)

## Abstract

**Contents:** Danshen is a popular herb employed to treat cardiovascular and cerebrovascular diseases worldwide. Danshen–drug interaction has not been well studied.

**Objective:** The inhibitory effects of four major tanshinones (tanshinone I, tanshinone IIA, cryptotanshinone, and dihydrotanshinone I) on UDP-glucuronosyltransferases (UGTs) isoforms were determined to better understand the mechanism of danshen–prescription drugs interaction.

**Materials and methods:***In vitro* recombinant UGTs-catalyzed 4-methylumbelliferone (4-MU) glucuronidation reaction was employed. Tanshinones (100 μM) was used to perform the initial screening of inhibition capability. High-performance liquid chromatography (HPLC) was used to separate 4-MU and its glucuronide. *In vitro–in vivo* extrapolation (IV–IVE) was employed to predict *in vivo* inhibition situation.

**Results:** Cryptotanshinone inhibited UGT1A7 and UGT1A9 with IC_50_ values of 1.91 ± 0.27 and 0.27 ± 0.03 μM, respectively. Dihydrotanshinone I inhibited UGT1A9-catalyzed 4-MU glucuronidation reaction with the IC_50_ value of 0.72 ± 0.04 μM. The inhibition of cryptotanshinone towards UGT1A7 and UGT1A9 was best fit to competitive inhibition type, and UGT1A9 was non-competitively inhibited by dihydrotanshinone I. Using *in vitro* inhibition kinetic parameters (*K_i_*) and *in vivo* maximum plasma concentration (*C*_max_) of cryptotanshinone and dihydrotanshinone I, the change of area-under-the-concentration–time curve (AUC) was predicted to be 0.4–4.2%, 3.7–56.3%, and 0.6–6.4% induced by cryptotanshinone and dihydrotanshinone inhibition towards UGT1A7 and UGT1A9, respectively.

**Discussion and conclusion:** The inhibitory effects of tanshinones towards important UGT isoforms were evaluated in the present study, which provide helpful information for exploring the mechanism of danshen–clinical drugs interaction.

## Introduction

Danshen, the dried root of *Salvia miltiorrhiza* Bunge, has been widely utilized to treat cardiovascular and cerebrovascular diseases all over the world, including China, Japan, the United States of America (USA), and European countries (Wang et al., 2007). Statistical data showed that the consumption of danshen-containing products costs $140 million since 2002 in USA (Lu et al., 2008). As one of the most popular herbal medicines, danshen has been always administered in combination with clinical drugs, possibly inducing severe herb–drug interaction (Tan et al., 2014). For example, danshen can increase the international normalized ratio of coagulation time and the risk of bleeding in patients administered warfarin through decreasing the elimination of warfarin (Chan et al., 1995). Additionally, co-treatment of danshen significantly increased the clearance of tolbutamide (Wang et al., 2010a–c).

In previous reports (Li et al., 2008; Qiu et al., 2008; Wang et al., 2010a–c), efforts have been made to explain the danshen–clinical drugs interaction; major components of danshen tanshinones are regarded as the substance basis for danshen–drug interaction. Influence of P-glycoprotein (P-gp) and cytochrome P450 (CYP) by these tanshinones is considered to be a potential reason for danshen–drug interaction. The experiment performed by Li et al. (2008) showed that tanshinone I was a substrate and an inhibitor of P-gp. Tanshinone I, IIA, dihydrotanshinone I, and cryptotanshinone exhibited the inhibitory effect towards human CYP1A2, 2C9, 2E1, and 3A4 with different inhibition modes (Qiu et al., 2008; Wang et al., 2010a–c).

UDP-glucuronosyltransferases (UGTs), important phase II enzymes, are responsible for catalyzing the glucuronidation reaction by adding the glucuronosyl group of UDP-glucuronic acid (UDPGA) as a cofactor (King et al., 2000). UGTs can catalyze the ∼35% of all drugs metabolized by Phase II enzymes (Evans & Relling, 1999). UGTs can also conjugate various endogenous substances including bilirubin, steroid hormones, thyroid hormones, bile acids, and fat-soluble vitamins (Ritter, 2000; Tukey & Strassburg, 2000). Inhibition of UGTs mediated reactions could not only result in serious drug–drug interactions (DDIs) but also induce metabolic disorders of endogenous substances. For example, indinavir, an HIV therapeutic drug, can significantly induce the elevation of unconjugated bilirubin in serum through inhibition of UGT1A1-mediated bilirubin glucuronidation (Zucker et al., 2001). Inhibition of UGT1A1 by sorafenib has been speculated to be main reason for sorafenib-induced elevation of serum bilirubin (Meza-Junco et al., 2009). Therefore, the inhibition of UGT activities by drugs should receive more attention from a clinical point-of-view. Many drugs and herbal constituents have been demonstrated to exhibit inhibition towards various UGT isoforms (Dong et al., 2012; Huang et al., 2010).

*In vitro* and *in vivo* experiments have indicated that predominant metabolic pathway for tanshinone IIa (TSA) is the NAD(P)H:quinone oxidoreductase 1 (NQO1)-mediated quinone reduction and subsequent glucuronidation, and UGT1A9 plays an important role in this process (Wang et al., 2010a–c). These results suggested that tanshinones can interact well with UGT isoforms, and the inhibitory effects of tanshinones towards UGT isoforms were speculated. Therefore, the aim of the present study is to investigate the inhibitory effects of four tanshinone components of danshen extract on catalytic activity of UDP-glucuronosyltransferases (UGTs). Detailed inhibition kinetic type and parameters were determined, and *in vitro–in vivo* extrapolation (IV-IVE) were performed to predict possible danshen-drug interactions due to the inhibition of UGT isoforms by tanshinones.

## Materials and methods

### Chemicals and reagents

Tanshinone I, tanshinone IIA, cryptotanshinone, and dihydrotanshinone I (purity ≥98%) were purchased from Aladdin Corp. (Shanghai, China). 4-Methylumbelliferone (4-MU), 4-methylumbelliferone-β-D-glucuronide (4-MUG), Tris-HCl, 7-hydroxycoumarin, and uridine 5′-diphosphoglucuronic acid (UDPGA) (trisodium salt) were purchased from Sigma-Aldrich (St Louis, MO). Recombinant human UGT supersomes (UGT1A1, UGT1A3, UGT1A6, UGT1A7, UGT1A8, UGT1A9, UGT1A10, UGT2B7, and UGT2B15) expressed in baculovirus-infected insect cells were obtained from BD Gentest Corp. (Woburn, MA). All other reagents were of HPLC grade or of the highest grade commercially available.

### Initial screening of the inhibitory potential of tanshinones towards various UGT isoforms

The probe substrate for all the UGT isoforms is 4-MU which is a non-selective substrate of UGTs. The incubation and analysis conditions were performed as previously described (Dong et al., 2012; Huang et al., 2010). The incubation mixture (200 μL) contained recombinant UGT isoforms (final concentration: 0.25, 0.05, 0.025, 0.05, 0.05, 0.05, 0.05, 0.05, and 0.75 μM for UGT1A1, UGT1A3, UGT1A6, UGT1A7, UGT1A8, UGT1A9, UGT1A10, UGT2B7, and UGT2B15, respectively), 5 mM UDPGA, 5 mM MgCl_2_, 50 mM Tris-HCl buffer (pH = 7.4), and 4-MU in the absence or presence of different concentrations of danshen's components. The concentrations of 4-MU were as follows: 110 μM for UGT1A1, 1200 μM for UGT1A3, 110 μM for UGT1A6, 15 μM for UGT1A7, 750 μM for UGT1A8, 30 μM for UGT1A9, 80 μM for UGT1A10, 350 μM for UGT2B7, and 250 μM for UGT2B15. The compounds were dissolved in DMSO, and the final concentration of DMSO was 0.5% (v/v). After 5 min pre-incubation at 37 °C, the UDPGA was added in the mixture to initiate the reaction. The incubation time was 120 min for UGT1A1, UGT1A10, UGT2B7, and UGT2B15, 75 min for UGT1A3, 30 min for UGT1A6, UGT1A7, UGT1A8, and UGT1A9. The reactions were quenched by adding 100 μL acetonitrile with 7-hydroxycoumarin (100 μM) as an internal standard. The mixture was centrifuged at 20,000 × *g* for 10 min and an aliquot of supernatant was transferred to an auto-injector vial for HPLC analysis. The HPLC system (Shimadzu, Kyoto, Japan) contained a SCL-10A system controller, two LC-10AT pumps, a SIL-10A auto injector, a SPD-10AVP UV detector. Chromatographic separation was carried out using a C18 column (4.6 × 200 mm, 5 μm, Kromasil) at a flow rate of 1 mL/min and UV detector at 316 nm. The mobile phase consisted of acetonitrile (A) and H_2_O containing 0.5% (v/v) formic acid (B). The following gradient condition was used: 0–15 min, 95–40% B; 15–20 min, 10% B; 20–30 min, 95% B. The calculation curve was generated by peak area ratio (4-MUG/internal standard) over the concentration range of 4-MUG 0.1–100 mM. The curve was linear over this concentration range, with *r*^2^ value >0.99. The limits of detection and quantification were determined at signal-to-noise ratios of 3 and 10, respectively. The accuracy and the precision of the back-calculated values for each concentration were less than 5%.

### Determination of inhibition kinetic parameters (*K_i_*)

The half-inhibition concentration (IC_50_) was determined as previously described (Fang et al., 2010). Inhibition kinetic parameters (*K_i_*) were determined utilizing various concentrations of 4-MU in the presence of different concentrations of components. Dixon and Lineweaver–Burk plots were adapted to determine the inhibition type, and the second plot of slopes from Lineweaver–Burk plot versus compound concentrations was utilized to calculate the *K_i_* value.

### *In vitro–in vivo* extrapolation (IV-IVE)

Equation (1) was utilized to predict the *in vivo* inhibitory magnitude of tanshinones towards UGTs-catalyzed glucuronidation reactions as previously described.

(1) $$\frac{\mathrm{AUCi}}{\mathrm{AUC}}=\frac{1}{1-f_{m}+\frac{f_{m}}{1+\frac{\left[I\right]}{K_{i}}}}$$

The terms are defined as followed: AUC_i_/AUC is the predicted ratio of *in vivo* exposure of drugs with co-administration of danshen's major ingredients. *f_m_* is the portion of the total clearance of drugs to which UGT isoforms contribute. *K_i_* is the reversible inhibition kinetic parameter. [*I*] is the *in vivo* concentration of major components of danshen.

## Results

### Tanshinones exhibited structure-dependent inhibition towards UGT isoforms

**Figure 1.**
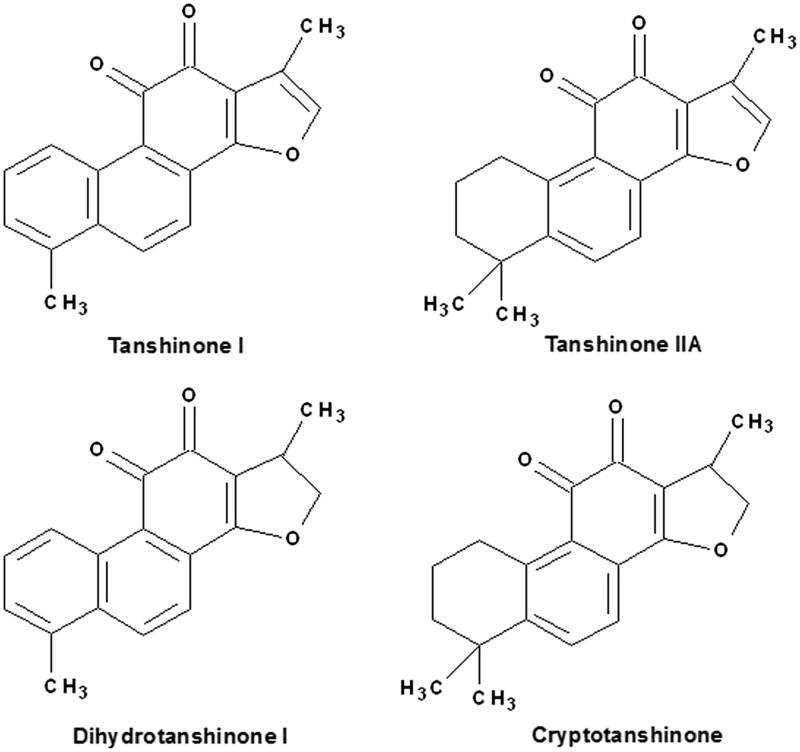
The structures of tanshinone I, tanshinone IIA, cryptotanshinone, and dihydrotanshinone I.

The structures of danshinones tested in the present study are shown in Figure 1. The inhibitory potential of tanshinones towards the various UGT isoforms is shown in Table 1. The results showed the influence of subtle differences of structures towards the inhibitory potential of tanshinones towards UGT isoforms. For example, the inhibition of cryptotanshinone and dihydrotanshinone I towards all UGT isoforms was stronger than tanshinone IIA and tanshinone I, respectively. That indicated that the addition reaction in the double bond of furan ring could increase the inhibition potential towards UGT isoforms.

**Table 1.** Initial screening of the inhibitory effects of danshen's major components towards UGTs. The values are given as mean plus SD (*n* = 3).

	Tanshinone I	Tanshinone IIA	Cryptotanshinone	Dihydrotanshinone I
UGT1A1	77.2 ± 2.8	69.8 ± 1.3	43.5 ± 1.2	67.3 ± 17.5
UGT1A3	68.2 ± 3.4	46.7 ± 1.6	22.2 ± 0.0	38.5 ± 0.4
UGT1A6	42.7 ± 0.5	38.7 ± 14.2	13.7 ± 3.4	38.9 ± 0.4
UGT1A7	25.8 ± 2.2	24.9 ± 2.4	7.2 ± 0.7	22.5 ± 9.3
UGT1A8	97.5 ± 1.7	79.7 ± 3.0	33.1 ± 7.6	52.1 ± 0.4
UGT1A9	29.9 ± 0.6	14.7 ± 3.1	4.9 ± 0.3	8.0 ± 0.0
UGT1A10	74.6 ± 2.2	56.8 ± 1.6	13.3 ± 4.8	37.2 ± 0.0
UGT2B7	67.6 ± 2.9	58.3 ± 0.2	12.8 ± 0.5	44.1 ± 6.6
UGT2B15	58.1 ± 0.8	65.6 ± 5.2	14.0 ± 0.5	28.6 ± 0.4

### The half inhibition concentration (IC_50_) of cryptotanshinone and dihydrotanshinone I

**Figure 2.**
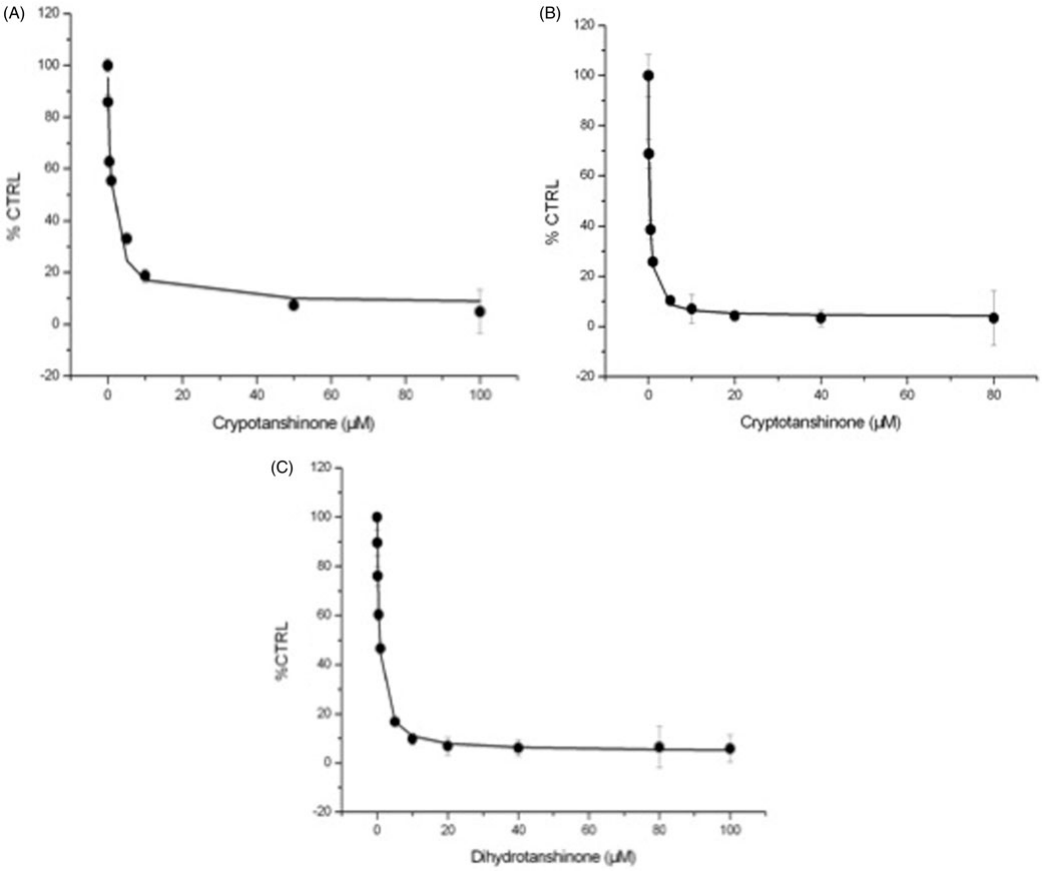
Determination of the half inhibition concentration (IC_50_). (A) The half inhibition concentration (IC_50_) of cryptotanshinone towards UGT1A7. (B) The half inhibition concentration (IC_50_) of cryptotanshinone towards UGT1A9. (C) The half inhibition concentration (IC_50_) of dihydrotanshinone I towards UGT1A9. Every data point represents the mean of two replicates.

The IC_50_ value was determined for the UGT isoforms whose activities were inhibited by more than 90% (Figure 2). The results showed that cryptotanshinone-inhibited UGT1A7 and UGT1A9 in a dose-dependent manner with IC_50_ values of 1.91 ± 0.27 and 0.27 ± 0.03 μM, respectively. Dihydrotanshinone I also inhibited UGT1A9-catalyzed 4-MU glucuronidation reaction in a concentration-dependent manner with the IC_50_ value of 0.72 ± 0.04 μM.

### Determination of inhibition kinetic type and parameters

**Figure 3.**
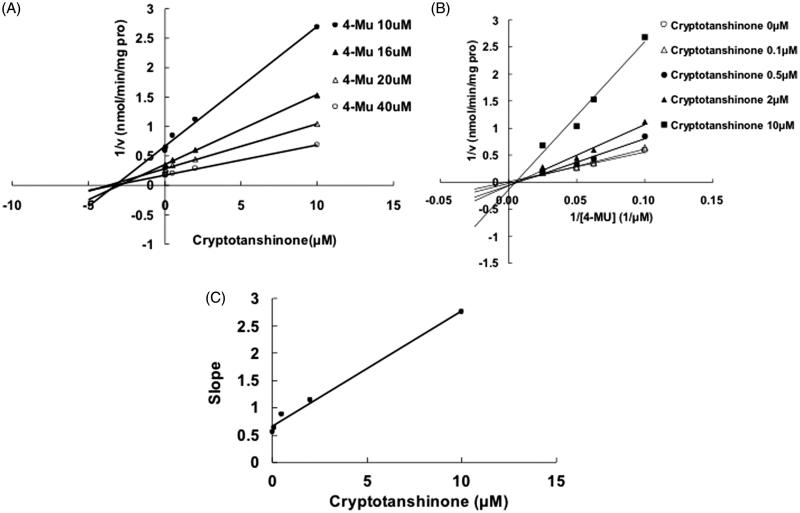
Inhibition of UGT1A7-mediated 4-MU glucuronidation reaction by cryptotanshinone. (A) Dixon plot of inhibitory effects of cryptotanshinone towards recombinant UGT1A7-catalyzed 4-MU glucuronidation. (B) Lineweaver–Burk plot of inhibitory effects of cryptotanshinone towards recombinant UGT1A7-catalyzed 4-MU glucuronidation. (C) Second plot of slopes from Lineweaver–Burk plot versus cryptotanshinone concentrations. Every data point represents the mean of two replicates.

**Figure 4.**
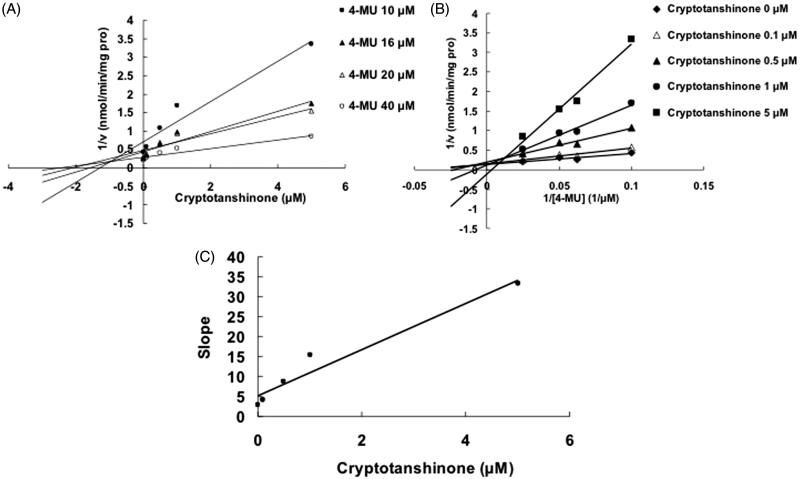
Inhibition of UGT1A9-mediated 4-MU glucuronidation reaction by cryptotanshinone. (A) Dixon plot of inhibitory effects of cryptotanshinone towards recombinant UGT1A9-catalyzed 4-MU glucuronidation. (B) Lineweaver–Burk plot of inhibitory effects of cryptotanshinone towards recombinant UGT1A9-catalyzed 4-MU glucuronidation. (C) Second plot of slopes from Lineweaver–Burk plot versus cryptotanshinone concentrations. Every data point represents the mean of two replicates.

**Figure 5.**
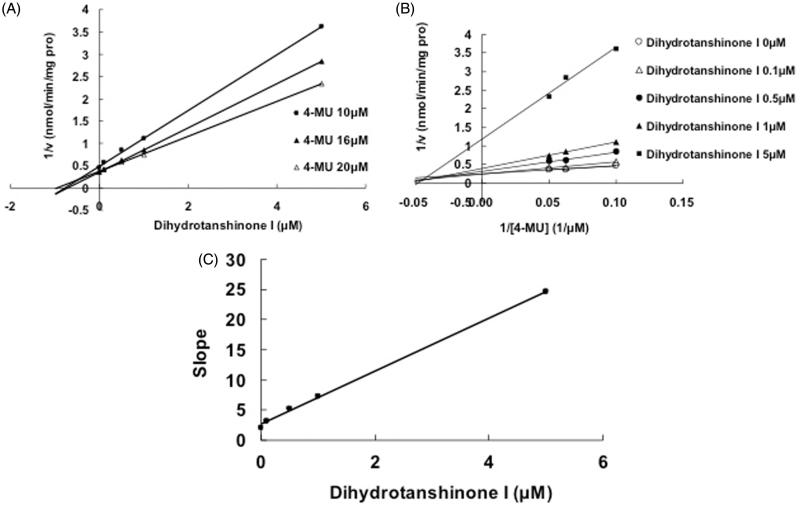
Inhibition of UGT1A9-mediated 4-MU glucuronidation reaction by dihydrotanshinone I. (A) Dixon plot of inhibitory effects of dihydrotanshinone I towards recombinant UGT1A9-catalyzed 4-MU glucuronidation. (B) Lineweaver–Burk plot of inhibitory effects of dihydrotanshinone I towards recombinant UGT1A9-catalyzed 4-MU glucuronidation. (C) Second plot of slopes from Lineweaver–Burk plot versus dihydrotanshinone I concentrations. Every data point represents the mean of two replicates.

Selecting different concentrations of cryptotanshinone or dihydrotanshinone I, the inhibition type and kinetic parameters were determined. The linear fitting was carried out using Dixon and Lineweaver–Burk equation. Figure 3(A) and (B) indicates the competitive inhibition of cryptotanshinone towards UGT1A7. The second plot (Figure 3C) using a slope from Lineweaver–Burk plot versus cryptotanshinone concentrations was used to determine the inhibition kinetic parameter (*K_i_*), and the value was calculated to be 3.1 μM. The Dixon plot (Figure 4A) and the Lineweaver–Burk plot (Figure 4B) indicated that cryptotanshinone competitively inhibited the UGT1A9-catalyzed 4-MU glucuronidation, and the *K_i_* value was calculated to be 0.25 μM. The inhibition of UGT1A9 by dihydrotanshinone I was best fit to noncompetitive type (Figure 5A and B), and the *K_i_* value was 0.61 μM.

### Prediction of *in vivo* danshen–drugs interaction

**Figure 6.**
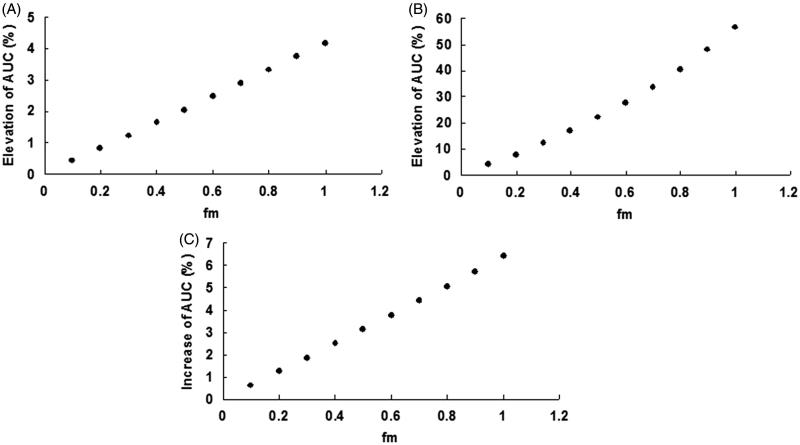
The curve of AUC*_i_*/AUC versus *f_m_*_._ (A) The *f_m_*-dependent change of AUC_i_/AUC due to the inhibition of cryptotanshinone towards UGT1A7. (B) The *f_m_*-dependent change of AUC_i_/AUC due to the inhibition of cryptotanshinone towards UGT1A9. (C) The *f_m_*-dependent change of AUC_i_/AUC due to the inhibition of dihydrotanshinone I towards UGT1A9.

In the present study, the maximum plasma concentration (*C*_max_) of cryptotanshinone and dihydrotanshinone I after administration of a mixture of danshen components for rats was used to predict the *in vivo* danshen–drugs interaction. The *C*_max_ was reported to be 42.85 ng/mL (0.14 μM) and 11.29 ng/mL (0.04 μM) for cryptotanshinone and dihydrotanshinone I, respectively. Besides *in vivo* concentrations of danshen components, the alteration of AUC for co-administered drugs also depends on the contribution of UGT isoforms (*f_m_*) towards the metabolism of drugs. So, the relationship between AUC's alteration and *f_m_* is described in Figure 6. For the inhibition of UGT1A7 by cryptotanshinone, the change of AUC ranged from 0.4% to 4.2%. Given that UGT1A7 was located into gastrointestine and the cryptotanshinone concentration which UGT1A7 was exposed to, the AUC change will be higher than the predicted value. For the inhibition of UGT1A9 by cryptotanshinone, the change of AUC ranged from 3.7% to 56.3%. For the inhibition of UGT1A9 by dihydrotanshinone I, the change of AUC ranged from 0.6% to 6.4%.

## Discussion

As a kind of famous herb, danshen has been widely used in China, and numerous pharmaceutical dosage forms can be commercially available, such as tablets, capsules, granules, injectables, oral liquids, sprays, and dripping pills (Chu et al., 2011). The most used product Fufang Danshen Dripping Pill has also cleared the US FDA phase II clinical trials, and will become the first Traditional Chinese Medicine product to obtain the drug approval. In view of the bright market prospects as well as keen interest in the modernization of herbal products, the safety problem should be clarified. Danshen-prescription drugs interaction has been frequently reported, however, the knowledge about its detailed mechanisms remains incomplete.

In recent years, the important role of UGT enzymes in the metabolic elimination of drugs or their phase I metabolites has been increasingly deciphered, which will provide more opportunities for deeply understanding clinical drug (herb)–drug interaction or drug-induced metabolic disorders of endogenous substances (Dong et al., 2012). For example, the HIV therapeutic drug indinavir can induce the elevation of serum-unconjugated bilirubin through inhibition of UGT1A1-mediated bilirubin glucuronidation (Zucker et al., 2001). Sorafenib-induced UGT1A1 inhibition might also be an important reason for the elevation of serum bilirubin (Meza-Junco et al., 2009). In contrast with the relatively complete understanding of the interaction between tanshinones and cytochrome P450 (CYP), the information on the interaction between tanshinones and UGT isoforms is limited.

UGT1A7, one of the core members of gastrointestinal UGT isoforms, plays an important role in the elimination of many xenobiotics through oral administration. For example, the highly carcinogen metabolites of tobacco benzo[a]pyrene (BaP) were demonstrated to be good substrates of UGT1A7 (Fang et al., 2002). Previous literature has shown that the alteration of UGT1A7 activity is a key factor to determine the cancer risk. Lu et al. (2011) performed meta-analysis to demonstrate that there is a cancer risk associated with intermediate and low activity UGT1A7 genotypes. UGT1A9, one of the most abundant UGT isoforms in the liver, is responsible for glucuronidation of various important clinical drugs, such as almokalant, flavopiridol, acetaminophen, tolcapone, and entacapone. Reduced activity of UGT1A9 often induces the adverse effects of drugs, such as the problematic for patients undergoing cancer treatment using irinotecan/SN-38 resulted from the low activity of UGT1A9. Therefore, the strong inhibition of cryptotanshinone and dihydrotanshinone I towards UGT1A7 and UGT1A9 should be given much attention.

The explanation of these *in vitro* data should be viewed with greater caution. First, herbs are extremely complex, and many factors can affect the quantities of herbal components, such as processing of herbs and environmental factors (soil, altitude, seasonal variation in temperature, length of daylight, rainfall pattern, shade, and dew) (Fang et al., 2011). Second, individual difference of pharmacokinetic factors (absorption, distribution, metabolism, and excretion) will affect the *in vivo* exposure dose of tanshinones. Finally, biphasic effect of herbs (short-term inhibition and long-term induction) should be considered to be another important factor influencing the final *in vivo* herb–drug interaction results. Previous studies have that the initial oral dose of *Schisandra* lignan extract (SLE) leads to an apparent inhibition towards CYP3A; however, its long-term consumption exhibits significant induction of CYP3A (Lai et al., 2009). The experiment performed by Ueng et al. showed that tanshinone IIA induced the activity of CYP1A, but exerted no effects towards UDP-glucuronosyltransferase (UGT) and glutathione *S*-transferase (GST). Further *in vivo* experiments will be performed to evaluate the net effects of inhibition and induction of drug-metabolizing enzymes by danshen's components.

In conclusion, the inhibitory effects of tanshinones towards important UGT isoforms were evaluated in the present study, which provide helpful information for exploring the mechanism of danshen–clinical drugs interaction. Given that the complication when using these *in vitro* data to extrapolate *in vivo* situation, more *in vivo* experiments should be carried out in the future.
